# Supplementary material for: Breast and cervical cancer screening among women at reproductive age in Cambodia: A secondary analysis of Cambodia Demographic and Health Survey 2022
Source: PLoS One. 2025 Jan 30;20(1):e0295881. doi: 10.1371/journal.pone.0295881 (PMC11781645; doi:10.1371/journal.pone.0295881)
Supplement: Attachment — Submitted filename: Response to reviewers.docx [file pone.0295881.s002.docx]

**Authors’ point-by-point responses to the editor and reviewers**

Manuscript ID: PONE-D-23-39984

Manuscript Titled: Breast and cervical cancer screening among women at reproductive age in Cambodia: Data analysis of Cambodia Demographic and Health Survey 2022.

**Dear editor and reviewers,**

We would like to thank all the reviewers and editor for providing their important time and effort in reviewing our manuscript. We have addressed all comments where relevant, as listed below:

**Reviewer #1:**

**Abstract:** The abstract could be outline as background, method, result and conclusion formats

**Author's response**: The abstract was written to comply with the requirements for PLOS ONE format.

Q1: Which one is one-third of all cancers worldwide? Is breast cancer or cervical cancer

**Authors’ response:** We revised it and highlighted in the revised manuscript accordingly.

Q2: Is demographic and health survey data incorporate behavioral factors that influence screening services? If so Which behavioural factors were included?

**Author’s response**: Behavioral factors that influence screening of breast self-examination and cervical screening services were not included in the CDHS 2022 questionnaire.

Q3: From which baseline the proportion of screening is low?

**Author's response**: We compared with DHS in other countries, such as *Nepal Demographic and Health Survey in 2022 and Kenya Demographic and Health Surveys in 2022*.

Q4: is media exposure or access to media data were collected by DHS survey?

**Authors’ response:** For clarity, the CDHS collected women's exposure to some types of media, such as *“Frequency of reading newspapers or magazines,*” *“Frequency of listening to radio,” and “Frequency of watching television.”* We have revised and *highlighted (Lines 162-167, Page 6).*

**Introduction:**

Q1: the national breast cancer and cervical cancer screening strategy, the current Cambodian policy about breast cancer and cervical cancer screening?

**Author’s response:** We have updated and highlighted the revised manuscript by adding *a National Action Plan for Cervical Cancer Prevention and Control 2019-2023. It has integrated HPV vaccination into the national immunization program to support cervical cancer prevention in Cambodia.* *Also, the new national action plan has addressed the screening methods for breast cancer included breast self-examination, physical examination and mammography. .*

Q2: Previous literatures about factors of breast cancer and cervical cancer screening?

**Authors’ response:** We have revised and highlighted in the text accordingly (Line 91-96)

Q3: What is your theoretical framework of this study and briefly put your theoretical framework

**Authors’ response:** It has been based on the social determinants of health. We have revised and highlighted in the text Line 97-99.

**Methods**

Q1: 254 reproductive age women were missed; how did you manage this missing data?

**Authors’ response:** The 254 women who reported “don’t known” were excluded from our analysis.

Q2: Has a doctor or other healthcare worker ever tested you for cervical cancer is your outcome question, if the women tested twice, three times, which one did you take?

**Author’s response:** in DHS, the women were ever asked for breast and cervical cancer screening. We have revised and highlighted in the revised manuscript for clarity for readers.

Q3: The independent variables could be listed for each outcome variables since the two outcome variables are quite different.

**Authors’ response:** We do not agree with the reviewer since the same independent variables were used to assess association with the two outcomes.

Q4: In DHS data there is clustering is so how did you manage this clustering in logistic regression analysis (why you conduct multilevel analysis).

Author’s response: We do not conduct multilevel analysis. We just use multiple logistic regression analysis, accounting for the complex survey design, as many published papers do such as: *Chhea, C., et al. (2018) in PLOS ONE, Um, S., et al. (2023) in PLOS ONE, and Lamichhane, B., et al. (2024) in PLOS GLOBAL PUBLIC HEALTH.*

Q5: What is the specific value of variance inflation factor (VIF)?

**Author’s response:** The use of VIF is to evaluate collinearity among independent variables. We have revised and highlighted the text accordingly in the revised manuscript (Line 167-170).

Q6: In one area it says logistic regression but in one area it says Ordinary Least Squares regression model, which one is correct?

**Author’s response:** The main analysis is the multivariate logistic regression. Therefor**e,** to avoid confusion for readers**,** we have revised that paragraph and highlighted accordingly. (Line 169-170)

Q7: how did you check model fitness?

**Author’s response: See**  our responses in Q5-Q6)

Q8: Is 19242 the sample size of both breast cancer and cervical cancer, if so how did you merge since the two outcomes were measured differently and it could have different missed value, different response and such like…please put the sample size of each outcome variables

**Author’s response:** Both outcome variables had the same samples collected. We have addressed this in Table 2. .

Q9: Operational definition of some independent variables could be necessary like wealth index, media exposure…..

**Authors’ response:** We have revised and highlighted in the revised manuscript accordingly.

Q10: Is method of screening and frequency of screening not your concern?

**Author’s response:** Yes, we do such as due to recall bias and social desirability. Therefore, we address these in our limitation.

Q11: is media access and media exposure the same in your study?

**Authors’ response:** We clarified this in Q4 abstract section and we have revised it to be consistently over the manuscript where relevant

Q12: What about the women already positive for breast cancer, and cervical caner and women age above 49 years?

**Authors’ response:** The DHS just collected data about ever breast cancer and cervical cancer screening in women aged 15-49 years old. As far as my knowledge, DHS did not collect data about breast and cervical cancer status.

**Results**

Q1: The mean age were calculated but reported with standard deviation why?

**Authors’ response:** In descriptive statistics, continuous variables such women's age was calculated as mean and SD. We have revised this in the text.

Q2: In your method section, you wrote that 19242 women were included for the final analysis, while in the result section, 19251 women were included. Why does this discrepancy occur (19242 VS 19251)?

**Authors’ response:** 19242 women were observed numbers, and 19251 women were weighted frequency used in DHS. For consistency, we have revised this to reflect our analysis and highlighted in Statistical analysis (Line 118-119).

Q3: How smoking was measured (smoking in last ten years, is smoking last 20 years or smoking on data collection) which one?

**Authors’ response:** In DHS, the smoking variable was asked as “*Current tobacco use status at the time of survey*”.

Q4: In alcohol drinking you classify never drink, ever drink and currently drink if so currently drink is included in ever drink individuals, how did you manage it?

**Authors’ response:** Current drink included only at least one drink in the last month.

Q5: You calculated Odds ratio but you say risk factor, how?

**Authors’ response:** We revised it accordingly, as highlighted in the text.

Q6: There is new variable in your result section, how?

**Authors’ response:** We have already re-checked, and we do not include any new variable.

**Discussion**

Q1: You did not measure awareness about breast self-examination

**Author’s Response**: Awareness about breast self-examination is not included in the CDHS 2022 questionnaire.

Q2: Please write the implication of your finding?

**Authors’ response:** We revised it accordingly, as highlighted in the text.

**Conclusion**

Q1: write result specific conclusion and recommendation

**Authors’ response:** We revised and highlighted in the revised manuscript accordingly.

Write the authors contribution of the study

**Author’s Response**: We will do so followed the PLOS ONE guideline including funding, authors' contributions, data availability, and competing interests after the main manuscript.

**Reviewer #2:**

The manuscript is very interesting and contains significant information on breast and cervical cancer screening across the reproductive age group and it is worthy for publication.

However, there are few minor suggestions which I would like to suggest.

1. In tittle, I would suggest that replace the "Data analysis" as "A secondary analysis/ A statistical analysis".

**Authors’ response:** We thank the reviewer for this feedback. We revised it accordingly.

2. In Table-2, It is well understood with only Yes (%) out of total sample of breast and cervical cancer screening. Here you can add the chi-square value.

**Authors’ response:** We thank the reviewer for this feedback. We revised it accordingly.
