# Supplementary material for: Breast and cervical cancer screening among women at reproductive age in Cambodia: A secondary analysis of Cambodia Demographic and Health Survey 2022
Source: PLoS One. 2025 Jan 30;20(1):e0295881. doi: 10.1371/journal.pone.0295881 (PMC11781645; doi:10.1371/journal.pone.0295881)
Supplement: Attachment — Submitted filename: Comment to the authors.docx [file pone.0295881.s003.docx]

Dear esteemed editor,

Thank you for providing me with the opportunity to review this manuscript. I would like to extend my appreciation to the authors for submitting a revised version of their manuscript. However, I would like to request that the authors provide a more detailed and elaborative response to the comments and suggestions provided in the previous review. Totally I am not satisfied with the revision. While I appreciate the effort made by the authors in their revision, I found that the point-by-point response in the submission documents was insufficient in addressing all of the concerns raised in the previous review. Therefore, I would like to request that the authors provide a more detailed and comprehensive response, addressing each of the comments and suggestions in a clear and concise manner.

Furthermore, I would like to request that the authors pay careful attention to the language used in their revision. It is important that the manuscript be written in a clear and concise manner, with proper grammar and syntax. This will ensure that the manuscript is easy to read and understand for all readers.

Thank you for your attention to these matters. The current comments are raised section by section below.

Abstract

1. On method section the likelihood measures should be written.
2. On result section it says the pooled prevalence from which did you pooled as we know DHS is secondary data, please clarify how did you pooled, and is only prevalence is pooled or factor variables also pooled?
3. On conclusion you conduct research on Adherence, while you conclude IFAS why?

Introduction

IFAS is one of strategy for morbidity and maternal mortality reduction due anemia. There is many articles and many systematic reviews conducted in previously so briefly show what is methodological gap, empirical gap, evidence gaps and knowledge gaps you could be filled in your research.

Method

1. Why 9 countries since East Africa isnot only 9
2. Ethiopia had demographic survey of 2019, why 2016?
3. Variable measurement needs reference
4. Result need more elaboration, in one area it says determinate, while in title it says factor, is it similar?
5. Discusion add the implication
6. In conclusion IFAS compliance was limited to one-third of pregnant mothers, what does it mean?
7. Conclusion is putting the result, please revise the conclusion
8. In conclusion the reader expected the recommendation but there is no single sentence which recommend based on your findings, why?
9. Reference 1, 2,10,18, is not full
