# Supplementary material for: Breast and cervical cancer screening among women at reproductive age in Cambodia: A secondary analysis of Cambodia Demographic and Health Survey 2022
Source: PLoS One. 2025 Jan 30;20(1):e0295881. doi: 10.1371/journal.pone.0295881 (PMC11781645; doi:10.1371/journal.pone.0295881)
Supplement: Attachment — Submitted filename: Response to reviewers_R2.docx [file pone.0295881.s004.docx]

**Authors’ point-by-point responses to the editor and reviewers**

**Manuscript ID: PONE-D-23-39984R1**

**Breast and cervical cancer screening among women at reproductive age in Cambodia: A Secondary Analysis of Cambodia Demographic and Health Survey 2022**

PLOS ONE

Dear Editor and Reviewers,

We would like to express our sincere gratitude to the editor-in-chief, academic editor, and reviewers for the significant time and effort put into further reviewing our manuscript. Given the further comments from reviewer #1 in R1 related to our revised manuscript. We, therefore, recheck and revise our manuscript point-by-point, as raised by the reviewer, where relevant, and resubmit it for your consideration. We believe that our revision will be clearer and reconsidered. We are committed to re-address all relevant concerns.

Again, thank you for your consideration and giving us the opportunity to revise our manuscript. If you have any questions or concerns, please don’t hesitate to contact me, as the corresponding author, via e-mail (umsamnang56@gmail.com).

Kind regards,

Samnang Um, PhD

National Institute of Public Health

Phnom Penh, Cambodia

Dear Dr. Um,

Thank you for submitting your manuscript to PLOS ONE. After careful consideration, we feel that it has merit but does not fully meet PLOS ONE’s publication criteria as it currently stands. Therefore, we invite you to submit a revised version of the manuscript that addresses the points raised during the review process.
==============================
Please review your reference list to ensure that it is complete and correct. If you have cited papers that have been retracted, please include the rationale for doing so in the manuscript text, or remove these references and replace them with relevant current references. Any changes to the reference list should be mentioned in the rebuttal letter that accompanies your revised manuscript. If you need to cite a retracted article, indicate the article’s retracted status in the References list and also include a citation and full reference for the retraction notice.

Additional Editor Comments:

Thank you for submitting the revised manuscript. Although you have made efforts to address most of the reviewers' comments, they are not yet fully satisfied with the revisions. The reviewers have asked for additional explanations and further revisions in certain sections of the manuscript. They have also suggested that the manuscript undergo a thorough English language edit or professional proofreading to improve clarity and readability.

Please make sure that these concerns are fully addressed and that the manuscript is thoroughly proofread before your next submission. We look forward to receiving your revised manuscript.
Thank you.

==============================

Please submit your revised manuscript by Aug 16 2024 11:59PM. If you will need more time than this to complete your revisions, please reply to this message or contact the journal office at [plosone@plos.org](mailto:plosone@plos.org). When you're ready to submit your revision, log on to <https://www.editorialmanager.com/pone/> and select the 'Submissions Needing Revision' folder to locate your manuscript file.

Please include the following items when submitting your revised manuscript:

- A rebuttal letter that responds to each point raised by the academic editor and reviewer(s). You should upload this letter as a separate file labeled 'Response to Reviewers'.
- A marked-up copy of your manuscript that highlights changes made to the original version. You should upload this as a separate file labeled 'Revised Manuscript with Track Changes'.
- An unmarked version of your revised paper without tracked changes. You should upload this as a separate file labeled 'Manuscript'.

If you would like to make changes to your financial disclosure, please include your updated statement in your cover letter. Guidelines for resubmitting your figure files are available below the reviewer comments at the end of this letter.

If applicable, we recommend that you deposit your laboratory protocols in [protocols.io](http://protocols.io/) to enhance the reproducibility of your results. Protocols.io assigns your protocol its own identifier (DOI) so that it can be cited independently in the future. For instructions see: <https://journals.plos.org/plosone/s/submission-guidelines#loc-laboratory-protocols>. Additionally, PLOS ONE offers an option for publishing peer-reviewed Lab Protocol articles, which describe protocols hosted on [protocols.io](http://protocols.io/). Read more information on sharing protocols at <https://plos.org/protocols?utm_medium=editorial-email&utm_source=authorletters&utm_campaign=protocols>.

We look forward to receiving your revised manuscript.

Kind regards,

Dr Pijush Kanti Khan, Ph.D.
Academic Editor
PLOS ONE

Reviewers' comments:
Reviewer's Responses to Questions

**Comments to the Author**

1. If the authors have adequately addressed your comments raised in a previous round of review and you feel that this manuscript is now acceptable for publication, you may indicate that here to bypass the “Comments to the Author” section, enter your conflict of interest statement in the “Confidential to Editor” section, and submit your "Accept" recommendation.

Reviewer #1: (No Response)

Reviewer #3: All comments have been addressed

**Author's response**: Thank

2. Is the manuscript technically sound, and do the data support the conclusions?

The manuscript must describe a technically sound piece of scientific research with data that supports the conclusions. Experiments must have been conducted rigorously, with appropriate controls, replication, and sample sizes. The conclusions must be drawn appropriately based on the data presented.

Reviewer #1: Partly

Reviewer #3: Yes

**Author's response**: Thank

3. Has the statistical analysis been performed appropriately and rigorously?

Reviewer #1: Yes

Reviewer #3: Yes

**Author's response**: Thank

4. Have the authors made all data underlying the findings in their manuscript fully available?

The [PLOS Data policy](http://www.plosone.org/static/policies.action#sharing) requires authors to make all data underlying the findings described in their manuscript fully available without restriction, with rare exception (please refer to the Data Availability Statement in the manuscript PDF file). The data should be provided as part of the manuscript or its supporting information, or deposited to a public repository. For example, in addition to summary statistics, the data points behind means, medians and variance measures should be available. If there are restrictions on publicly sharing data—e.g. participant privacy or use of data from a third party—those must be specified.

Reviewer #1: No

Reviewer #3: Yes

**Author's response**: Thank

5. Is the manuscript presented in an intelligible fashion and written in standard English?

PLOS ONE does not copyedit accepted manuscripts, so the language in submitted articles must be clear, correct, and unambiguous. Any typographical or grammatical errors should be corrected at revision, so please note any specific errors here.

Reviewer #1: No

Reviewer #3: Yes

**Author's response**: Thank

6. Review Comments to the Author

Please use the space provided to explain your answers to the questions above. You may also include additional comments for the author, including concerns about dual publication, research ethics, or publication ethics. (Please upload your review as an attachment if it exceeds 20,000 characters)

Reviewer #1: Dear esteemed editor,
Thank you for providing me with the opportunity to review this manuscript. I would like to extend my appreciation to the authors for submitting a revised version of their manuscript. However, I would like to request that the authors provide a more detailed and elaborative response to the comments and suggestions provided in the previous review. Totally I am not satisfied with the revision. While I appreciate the effort made by the authors in their revision, I found that the point-by-point response in the submission documents was insufficient in addressing all of the concerns raised in the previous review. Therefore, I would like to request that the authors provide a more detailed and comprehensive response, addressing each of the comments and suggestions in a clear and concise manner.
Furthermore, I would like to request that the authors pay careful attention to the language used in their revision. It is important that the manuscript be written in a clear and concise manner, with proper grammar and syntax. This will ensure that the manuscript is easy to read and understand for all readers.
Thank you for your attention to these matters. The current comments are raised section by section below.
Abstract
1. On method section the likelihood measures should be written.

**Author's response**: This is not relevant questions

2. On result section it says the pooled prevalence from which did you pooled as we know DHS is secondary data, please clarify how did you pooled, and is only prevalence is pooled or factor variables also pooled?

**Author's response**: This is not relevant questions

3. On conclusion you conduct research on Adherence, while you conclude IFAS why?

**Author's response**: This is not relevant questions

Introduction
IFAS is one of strategy for morbidity and maternal mortality reduction due anemia. There is many articles and many systematic reviews conducted in previously so briefly show what is methodological gap, empirical gap, evidence gaps and knowledge gaps you could be filled in your research.

**Author's response**: This is not relevant questions

Method
1. Why 9 countries since East Africa isnot only 9

**Author's response**: This is not relevant questions

2. Ethiopia had demographic survey of 2019, why 2016?

**Author's response**: This is not relevant questions

3. Variable measurement needs reference

**Author's response**: This is not relevant questions

4. Result need more elaboration, in one area it says determinate, while in title it says factor, is it similar?

**Author's response**: This is not relevant questions

5. Discusion add the implication

**Author's response**: This is not relevant questions

6. In conclusion IFAS compliance was limited to one-third of pregnant mothers, what does it mean?

**Author's response**: This is not relevant questions

7. Conclusion is putting the result, please revise the conclusion

**Author's response**: This is not relevant questions

8. In conclusion the reader expected the recommendation but there is no single sentence which recommend based on your findings, why?

**Author's response**: This is not relevant questions

9. Reference 1, 2,10,18, is not full

**Author's response**: This is not relevant questions

Reviewer #3: Thank you for allowing me to review this interesting work. The authors have effectively responded to the comments. I recommend publishing this article after thorough proofreading.

**Author's response**: This is not relevant questions

7. PLOS authors have the option to publish the peer review history of their article ([what does this mean?](https://journals.plos.org/plosone/s/editorial-and-peer-review-process#loc-peer-review-history)). If published, this will include your full peer review and any attached files.


If you choose “no”, your identity will remain anonymous but your review may still be made public.


**Do you want your identity to be public for this peer review?** For information about this choice, including consent withdrawal, please see our [Privacy Policy](https://www.plos.org/privacy-policy).

Reviewer #1: No

Reviewer #3: No
